# Circulating cell-free mature microRNAs and their target gene prediction in bovine metritis

**DOI:** 10.1038/srep29509

**Published:** 2016-07-11

**Authors:** Vanmathy Kasimanickam, John Kastelic

**Affiliations:** 1 Veterinary Clinical Sciences Department & Center for Reproductive Biology, College of Veterinary Medicine, Washington State University, Pullman, WA 99164, USA; 2 Department of Production Animal Health, Faculty of Veterinary Medicine, University of Calgary, Calgary, AB T2N 4Z6, Canada

## Abstract

Uterine infections in dairy cows are common after calving, reduce fertility and cause substantial economic losses. Conventional diagnosis (based on clinical signs) and treatment can be challenging. Serum microRNA (miRNA) profiles serve as non-invasive biomarkers in several pathological conditions including inflammatory diseases. The objective was to identify differentially expressed serum miRNAs in cows with metritis and normal uterus (four cows per group), integrate miRNAs to their target genes, and categorize target genes for biological processes involved in bacterial infection and inflammatory responses. Out of 84 bovine-specific, prioritized miRNAs analyzed, 30 were differentially expressed between metritis and normal cows (p ≤ 0.05, fold regulation ≥2 magnitudes). Bta-miR-15b, bta-miR-17-3p, bta-miR-16b, bta-miR-148a, bta-miR-26b, bta-miR-101 and bta-miR-29b were highly up-regulated whereas bta-miR-148b, bta-miR-199a-3p, bta-miR-122, bta-miR-200b and bta-miR-10a were highly down-regulated in cows with metritis compared to cows with normal uterus. Highly scored target genes of up-regulated and down-regulated miRNAs were categorized for various biological processes, including biological regulation, cellular process, developmental process, metabolic process, localization, multicellular organismal process, response to stimulus, immune system process, cellular components organization, apoptotic process, biological adhesion, developmental process, and locomotion that are critical to combat bacterial infections and provoke inflammatory responses.

Uterine diseases are prevalent in dairy cows, cause poor reproductive performance, reduced milk yield, and substantial economic losses^1^,^2^,^3^. Uterine diseases can be classified as puerperal metritis, clinical metritis, clinical endometritis and subclinical endometritis^4^,^5^,^6^. Although uterine diseases are often diagnosed and classified by systemic signs, nature of uterine discharge, uterine cytology and systemic illness, diagnosis can be challenging^4^,^5^,^6^. Consequently, gene expression and protein production due to uterine inflammation in postpartum dairy cows have been studied^7^,^8^,^9^,^10^. Gene expression of key inflammatory cytokines such as tumor necrosis factor-α (TNF-α), interleukin (IL)-1β and IL-6 in blood monocytes varied between post-partum cows with metritis versus normal uterus^11^. Endometrial gene expression of mucin-1 (MUC-1) and various cytokines [Toll-like receptor (TLR) 4, IL-1β, IL-8, TNF-α, insulin-like growth factor-1 (IGF-1), and IGF-binding protein-2 (IGF-BP-2)] differed in cows with uterine inflammation compared to unaffected post-partum cows^12^. Although several studies have considered genetic components of uterine inflammation in dairy cattle^11^,^12^,^13^, few investigations have elucidated epigenetic changes such as altered expression of regulatory RNAs and their subsequent integration with coding genes that participate in bovine metritis^14^,^15^.

Epigenetics denotes heritable changes in gene expression that are not involved with the coding sequence modifications. These epigenetic alterations are manifested by DNA methylation, chromatin remodeling, histone modifications and small non-coding RNAs^16^. Mammalian microRNAs (miRNA) are small (20 to 25 nucleotides) evolutionarily conserved non-coding RNAs. They are transcribed by RNA polymerase II enzyme in the nucleus as a long primary transcript (pri-miRNA) which may contain more than one mature miRNAs. Subsequently, pri-miRNAs are processed by RNase III enzyme (e.g., Drosha-DGCR8 complex) to form pre-miRNAs, which are exported to the cytosol by exportin 5. In the cytoplasm, Dicer processes pre-miRNAs to mature miRNAs. Subsequently, one of the strands is incorporated with RNA-induced silencing complex (RISC) where it is directed to its target mRNAs^17^,^18^. The MiRNAs are involved in both post-transcriptional gene regulation (causes translational suppression) and direct degradation of mRNAs^19^. To date, 793 bovine mature miRNAs have been identified (http://www.mirbase.org) although their role in pathogenesis of disease is not well documented.

Means and mechanisms that regulate expression of inflammatory mediators and terminate their activities are important to understand pathogenesis of uterine inflammatory diseases due to various infectious agents. A broad range of regulatory roles of miRNAs in infectious and inflammatory diseases in humans have been investigated. Perturbations of miRNAs at tissue expression level and in peripheral circulation have been demonstrated. Induced miR-155 in macrophages potentiated the immune response against *Salmonella typhimurium* in mice vaccinated against this bacterium^20^. Furthermore, there were increased levels of intra-renal and urinary miR-155 and miR-146a in human IgA associated inflammatory nephropathy^21^; therefore, various miRNAs levels in body fluids might serve as non-invasive biomarkers in inflammatory diseases.

Regulatory roles of miRNAs in autoimmune diseases including diabetes, atopic dermatitis, Sjogren’s syndrome and inflammatory bowel disease have also been recognized^22^,^23^,^24^,^25^,^26^. Furthermore, regulatory functions of microRNAs in normal uterine physiological status and in pathological disorders (e.g., endometriosis, dysfunctional uterine bleeding and endometrial cancer) in humans have been addressed^27^. Potential regulatory role of miRNAs in development and progression of bovine subclinical endometritis has been investigated by studying expression of miRNAs in uterine endometrial samples^28^,^29^. The objective of this study was to identify differentially expressed serum miRNAs in cows with metritis or a normal uterus (four cows in each group), integrate miRNAs to their target genes, and categorize target genes for biological processes involved in bacterial infection and inflammatory responses.

## Materials and Methods

### Ethics statement

This study was performed in strict accordance with the ethics, standard operating procedure, handling of animals, collection of biomaterials, and use of biofluids for research. The protocol was approved by the institutional animal care and use committee of the Washington State University (Protocol Number: 04070-001).

### Cows and sample collection

Cows with metritis (n = 4) of similar severity and cows with no apparent uterine abnormality (n = 4) within 1 wk after calving were included. Affected cows had reddish brown fetid uterine discharge and elevated rectal temperature (>39.5 °C), whereas normal cows had clear or no uterine discharge and <18% neutrophils on uterine cytology at 21 d post-partum. Blood was collected by coccygeal venipuncture from these postpartum cows at the time of diagnosis and used to investigate circulating miRNAs.

### Blood sample processing

Silica spray-coated red-top 10 mL serum tubes (BD Biosciences, San Jose, CA, USA) were used to collect blood. Samples were allowed to clot for 15 min at room temperature and then put on ice in a cooler and transported to the laboratory within 2 h after collection. Tubes were centrifuged (1000 × g for 10 min) in a refrigerated centrifuge and serum was apportioned into 0.5-mL aliquots and stored at −80 °C until further processing.

### Small RNA isolation and reverse transcription

Small RNAs were purified using a miRNeasy serum/plasma kit (Qiagen, Valencia, CA, USA). The miRNeasy serum/plasma kit includes phenol/guanidine-based lysis of samples and silica membrane column-based isolation of small RNAs. The kit was designed to isolate cell-free small RNAs. Frozen serum samples were thawed at room temperature, 150 μL of serum was pipetted into a 1.5 mL eppendorf tube, and 750 μL of QIAzol reagent was added, mixed by repeated pipetting and incubated at room temperature for 5 min. Following incubation, 3.5 μL miRNeasy serum/plasma spike-in-controls (lyophilized C. elegans miR-39 miRNA mimic) at 1.6 × 10^8^ copies/μL was added. Then, 150 μL of chloroform was added and the mixture was shaken vigorously for 15 s, incubated at room temperature for 2–3 min, and then centrifuged at 12,000 × g for 15 min at 4 °C. The upper portion of the aqueous phase was transferred to a new 1.5 mL eppendorf tube without contaminating the interphase. Approximately 1.5 volumes of 100% ethanol was added to the aqueous contents and thoroughly mixed by pipetting. Approximately half of the mix was transferred into an RNeasy MinElute spin column in a 2 mL collection tube and centrifuged at 12,000 × g for 15 s at room temperature. The flow-through was discarded and this step was repeated with the rest of the sample. The RNA was bound to the membrane. Subsequently, membrane with attached RNAs was washed by 700 μL of buffer RWT and 500 μL of buffer RPE with two centrifugation steps. Then, the RNeasy MinElute column in a new 2 mL collection tube was dried for 5 min by a full speed centrifugation step (14,000 × g) and the flow-through and collection tube were discarded. Subsequently, small RNAs were eluted in a new 1.5 mL collection tube in 24 μL RNase-free water by a centrifugation step (14,000 × g for 1 min).

The RNA was reverse transcribed using miScript II RT kit (Qiagen, Valencia, CA, USA). A HiSpec buffer (5×) was used to prepare cDNA for mature miRNA profiling. RNase-free water, 10x miScript Nucleics mix and 5x HiSpec buffer were thawed at room temperature. All contents were mixed by flicking the tubes, spun them briefly to collect residues and kept on ice. A reverse transcription reaction was prepared by adding 4 μL of HiSpec buffer, 2 μL of Nucleics mix, 2 μL of miScript reverse transcriptase enzyme and 12 μL of the template RNA. The reaction was gently mixed and briefly spun. The mix was incubated at 37 °C for 60 min. Then, reverse transcriptase enzyme was inactivated by incubation at 95 °C for 5 min and the content was placed on ice. Subsequently, the cDNA was diluted in nuclease free water (final volume, 110 μL) and stored at −20 °C.

### Mature bovine miRNA expression profiles

MiScript miRNA PCR array technology was used to identify circulating cell-free mature miRNA in bovine serum. Since this expression profiling consists of SYBR green-based real-time PCR, single miRNA gene validation experiments were not considered necessary. A bovine miRBase profiler plate 1 (Table 1, Qiagen, Valencia, CA, USA) was used. The array plate consisted of specific primers to identify 84 highly prioritized bovine mature miRNAs from the most current miRNA genome, as annotated in miRBase V20 (www.miRBase.org). Controls included were cel-miR-39-3p, SNORD42B, SNORD69, SNORD61, SNORD68, SNORD96A, RNU6-2, miRTC and PPC.

MiScript SYBR Green PCR Kit (Qiagen, Valencia, CA, USA) which contains miScript Universal Primer (reverse primer) and QuantiTect SYBR Green PCR Master Mix, was used to prepare real-time PCR reactions. The reaction volume (2750 μL) was prepared using 1375 μL of Master Mix, 275 μL of Universal Primer, 1000 μL of RNase-free water and 100 μL of template cDNA, with 25 μL of reaction volume added to each well (96-well plates). Amplification was programmed in a StepOne Plus instrument (Applied Biosystems Inc., Carlsbad, CA, USA). Cycling conditions for real-time PCR included an initial activation step at 95 °C for 15 min to activate HotStar Taq DNA polymerase and 40 cycles of denaturation (at 94 °C for 15 s), annealing (at 55 °C for 30 s) and extension (at 70 °C for 30 s). The ROX passive reference dye was designed to normalize fluorescent reporter signal and baseline and threshold were set automatically for all real-time PCR runs. Fluorescence data were collected at the holding stage of the extension step. Specificity and identity were verified by melting curve analyses. Threshold cycles values (C_T_) were exported as an Excel file for analyses.

### Bovine mature miRNA PCR array analysis

Eighty-four high-priority bovine mature miRNAs were selected from the miRNA genome to analyze. Reverse transcription and positive controls were chosen to ensure efficiency of the array, reagents and instrument. Raw C_T_ in Excel version (1997–2003 (.XLS file format)) was uploaded to the data analysis center (http://www.qiagen.com). Data quality control was examined to assess amplification reproducibility and reverse transcription efficiency, and to detect any other contamination in amplified samples. The C_T_ values of samples were calibrated to the C_T_ values of cel-miR-39-3p. Global C_T_ mean of expressed miRNAs was chosen to normalize the target circulating miRNAs. The distribution of C_T_ values and raw data average in both groups were reviewed. Average ΔC_T_, 2^^−ΔCT^, fold change, P-value and fold regulation were calculated in the web-based program and P-values were included in subsequent graphical analyses. Average C_T_ values were converted into linear 2^^−ΔCT^ values and P-values were calculated with a Student’s *t*-test.

### Computational prediction of potential mRNAs targets

Target genes were predicted using miRDB (http://mirdb.org/miRDB/) for differentially expressed miRNAs in metritis, and top ranked predicted genes were run through a PANTHER classification system^18^ to identify associated biological processes in response to uterine infection and inflammation.

## Results and Discussion

**Figure 1:**
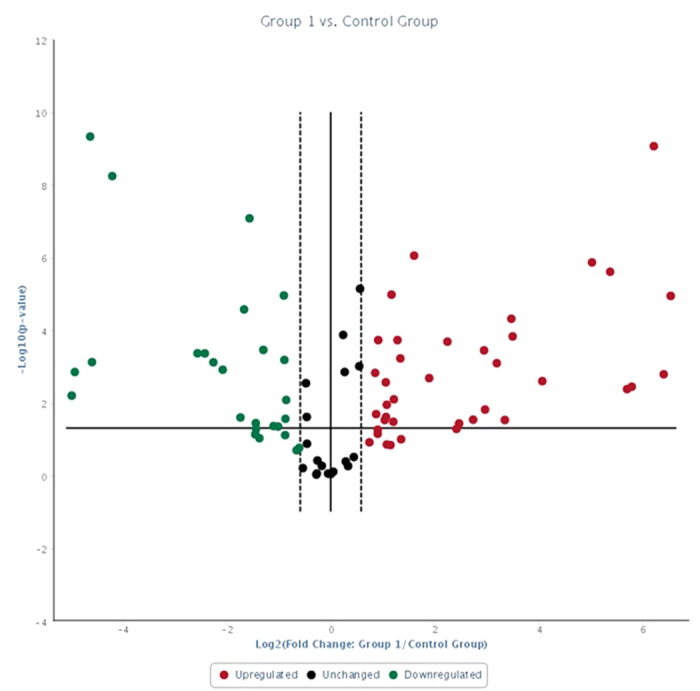
Volcano plot: Metritis versus control group. Boundary = 1.5; p < 0.05; Undetermined miRNAs in both groups were removed.

**Figure 2:**
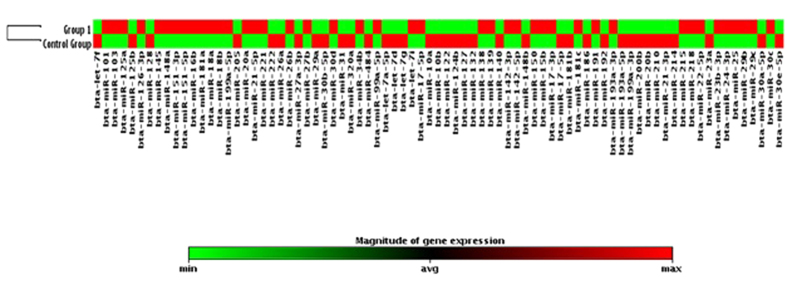
Clustergram of target miRNAs in metritis (Group 1) and control groups. p < 0.05; Undetermined miRNAs in both groups were removed.

On miRNA quantitative profiling, 30 miRNAs were differentially expressed (p ≤ 0.05; Fold Regulation ≥2 magnitude) in serum samples of cows with metritis compared to normal cows, out of 84 bovine specific miRNAs investigated (Figs 1 and 2). Highly up-regulated miRNAs were bta-miR-15b, bta-miR-17-3p, bta-miR-16b, bta-miR-148a, bta-miR-26b, bta-miR-101, bta-miR-29b, bta-miR-27b and bta-miR-215 (≥10 magnitude of Fold Regulation) whereas bta-miR-148b, bta-miR-199a-3p, bta-miR-122, bta-miR-200b and bta-miR-10a (≤−10 magnitude of Fold Regulation) were highly down-regulated in cows with metritis compared to control cows (Table 2). Response of host cells to microbial infection, and immune activation and inflammatory process by host cells in metritis might have caused dysregulation of miRNAs in the present study. In mice^30^, miR-15b was highly up-regulated in whole blood upon intra-peritoneal injection of lipopolysaccharide, a potent mediator of gram negative bacteria implicated in sepsis and inflammation.

Lipopolysaccharide complex associated with gram-negative pathogens (e.g., *Escherichia coli*) in cows with metritis may have contributed to up-regulation of bta-miR-15b in the current study. *Escherichia coli* is one among the main types of bacteria causing uterine inflammation, and the uterine pathology is largely caused with the bacterial endotoxin lipopolysaccharide. The highly expressed bta-miR-15b may have down-regulated its target genes that are necessary for the physiological events associated with the normal uterine involution. In that regard, bovine-specific miRNAs were differentially expressed in normal mammary tissue challenged with *Staphylococcus aureus*^31^ and differentially expressed miRNAs were bacteria-specific. Bta-miR-184, miR-24-3p, miR-148, miR-486 and let-7a-5p were unique to *E. coli*, whereas bta-miR-2339, miR-499, miR-23a and miR-99b were specific to *S. aureus* in an *in-vitro* study with bovine mammary epithelial cells^32^. In the present study, upregulation of bta-miR-148a may have been due to *E. coli* infection associated with metritis. Post-partum involution completes the reproductive cycle after pregnancy and calving by returning the uterus to its normal non-pregnant state so that the cow can come into estrus again. This uterine involution involves substantial uterine tissue reorganization via the activation of matrix metalloproteases, extracellular matrix degradation and cellular autophagy/apoptosis. This is driven by several cellular processes and multitude of gene expression. The *E. coli* infection may have down-regulated the genes necessary for the uterine involution through up-regulation of bta-miR-148a. In addition, some of the predicted target genes for the bta-miR-148 are involved in apoptosis and matrix degradation. MiR-101 regulated the innate immune response of macrophages challenged with lipopolysaccharide via its target gene MAPKP-1^33^. Serum levels of miR-16, miR-17, miR-20a, miR-20b, miR-26a, and miR-26b were up-regulated in an experimental sepsis condition induced by cecal ligation and puncture in mice^34^, whereas over-expression of miR-21, miR-29b and miR-148a occurred in systemic lupus erythematosus^35^,^36^. Differential expression of these miRNAs’ would have been an outcome of immune-mediated inflammatory responses in systemic lupus erythematosus, and in the present study, up-regulation of bta-miR-148a may be the result of uterine inflammatory processes in response to post-partum microbial infections. This would have been affected the normal uterine involution by repressing the genes involved in matrix degradation, cell autophagy and cell apoptosis.

Interestingly, in the current study, serum bta-miR-148b level was lower, in contrast to the higher serum level of bta-miR-148a in cows with metritis, although they belong to the same miRNA gene family. This might be plausible, since top-ranked target genes, based on miRDB total target score for both miRNAs were different, suggesting molecular functions and biological processes can diverge. Infection with *L. monocytogenes* significantly reduced expression of intestinal miR-200b^37^, whereas in the present study, bta-miR-200b was down-regulated, consistent with involvement of a bacterial pathogen or an inflammatory outcome of a comparable process in bovine metritis. Also, miR-200b was inversely correlated to transforming growth factor-beta 1(TGF-β1) and TGF-β family involves in preparation of pregnancy and in vascular remodeling. Therefore, repressed miR-200b may have activated the TGF-β signaling and may have caused the uterine inflammation and hindered the normal involution. MiR-122 has been considered liver-specific and associated with hepatitis C virus infection^38^. However, in the current study, the serum level of bta-miR-122 was highly divergent between cows with metritis and control cows, emphasizing the necessity of future exploration of its function with regards to metritis. Interestingly, miR-199a maintained uterine quiescence by suppressing COX-2, enhancing contractile prostaglandins, and mediating progesterone and estrogen effects during pregnancy and labor in humans^39^, whereas a lower level of serum bta-miR-199a in the current study may have contributed to clearance of infectious materials and inflammatory products from the bovine uterus. Regulatory T-cells control immune responses, and miR-10a was reported to be a key mediator of Treg in pathogen-mediated inflammation^40^, whereas serum bta-miR-10a was lower in cows with metritis in the current study, suggesting a critical role in the biological process of uterine inflammation.

**Figure 3:**
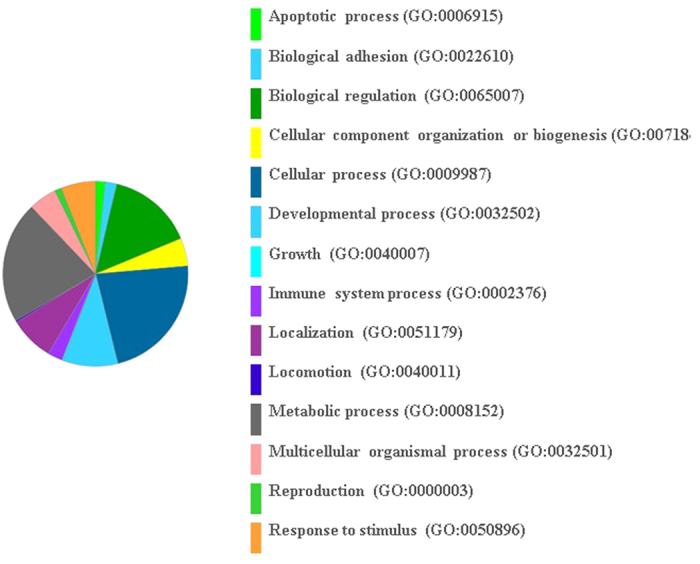
Integrated genes for up-regulated miRNAs in metritis. Go biological process; number of genes run into the gene list analysis in the PANTHER classification system was 200; number of genes hit was 172; total number of process hit was 347.

**Figure 4:**
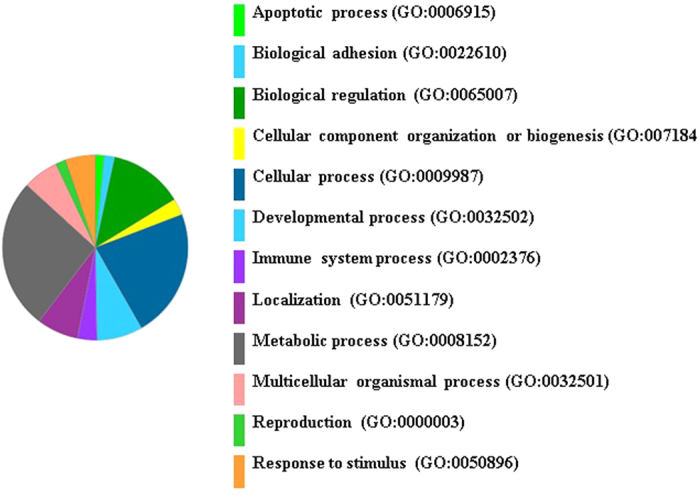
Integrated genes for down-regulated miRNAs in metritis. Go biological process; number of genes run into the gene list analysis in the PANTHER classification system was 200; number of genes hit was 196; total number of process hit was 386.

In the current study, target genes were identified (miRDB^28^) for highly differentiated miRNAs between metritis and control cows. Up-regulated miRNAs in metritis were integrated to several genes; top ranking genes were ZFHX4, SYNJ1, CDCA4, KAT7, TSHZ3, MAML3, ZFHX4, SLC9A6, and IPO7, whereas top-ranking genes with down-regulated miRNAs were DLST, ZC3H14, ETS1, ETNK1, CELSR2, ADAMTSL3, HNRNPU, CLIC5, and CLIC4 (Tables 3 and 4). On the analysis of 200 targeted genes of the 10 up-regulated miRNAs, and 200 integrated genes of the down-regulated miRNAs, using the PANTHER classification system, genes were categorized into several biological processes, including biological regulation, cellular process, developmental process, metabolic process, localization, multicellular organismal process, response to stimulus, immune system process, cellular components organization, apoptotic process, biological adhesion, developmental process, and locomotion (Figs 3 and 4). Several of these biological processes are critical in responses to uterine infection, for inflammatory progression, and for clearing infectious materials and inflammatory products following the course of metritis.

## Conclusion

In conclusion, there were highly discriminated serum miRNAs identified between cows with metritis and normal cows. Of 84 bovine-specific prioritized miRNAs investigated, 16 and 14 were present in higher and lower levels, respectively, in cows with metritis. Hundreds of integrated genes were identified for these up-regulated and down-regulated miRNAs, and top-ranked genes based on total target scores were categorized into biological processes critical for responding to uterine infection, mediating uterine inflammatory process, and for clearance of infectious agents and inflammatory products.

## Additional Information

**How to cite this article**: Kasimanickam, V. and Kastelic, J. Circulating cell-free mature microRNAs and their target gene prediction in bovine metritis. *Sci. Rep.* **6**, 29509; doi: 10.1038/srep29509 (2016).

## Figures and Tables

**Table 1:** Cow miRBase profiler plate 1, consisting of primers for 84 target miRNAs and primers for control genes.

	1	2	3	4	5	6	7	8	9	10	11	12
A	bta-let-7f	bta-miR-101	bta-miR-103	bta-miR-125a	bta-miR-125b	bta-miR-126-3p	bta-miR-128	bta-miR-145	bta-miR-148a	bta-miR-151-3p	bta-miR-151-5p	bta-miR-16b
B	bta-miR-181a	bta-miR-18a	bta-miR-18b	bta-miR-199a-5p	bta-miR-205	bta-miR-20a	bta-miR-21-5p	bta-miR-221	bta-miR-222	bta-miR-26a	bta-miR-26b	bta-miR-27a-3p
C	bta-miR-27b	bta-miR-29a	bta-miR-30b-5p	bta-miR-30d	bta-miR-31	bta-miR-320a	bta-miR-34b	bta-miR-484	bta-miR-499	bta-miR-99a-5p	bta-let-7a-5p	bta-let-7d
D	bta-let-7g	bta-let-7i	bta-miR-106a	bta-miR-107	bta-miR-10a	bta-miR-10b	bta-miR-122	bta-miR-124a	bta-miR-127	bta-miR-132	bta-miR-138	bta-miR-139
E	bta-miR-140	bta-miR-142-3p	bta-miR-142-5p	bta-miR-148b	bta-miR-150	bta-miR-15b	bta-miR-17-3p	bta-miR-17-5p	bta-miR-181b	bta-miR-181c	bta-miR-186	bta-miR-191
F	bta-miR-192	bta-miR-193a-3p	bta-miR-193a-5p	bta-miR-199a-3p	bta-miR-199b	bta-miR-200a	bta-miR-200b	bta-miR-200c	bta-miR-20b	bta-miR-210	bta-miR-21-3p	bta-miR-214
G	bta-miR-215	bta-miR-218	bta-miR-22-5p	bta-miR-23a	bta-miR-23b-3p	bta-miR-24-3p	bta-miR-25	bta-miR-29b	bta-miR-29c	bta-miR-30a-5p	bta-miR-30c	bta-miR-30e-5p
H	cel-miR-39-3p	cel-miR-39-3p	SNORD42B	SNORD69	SNORD61	SNORD68	SNORD96A	RNU6-6P	miRTC	miRTC	PPC	PPC

**Table 2:** Fold regulation of serum miRNAs in cows with metritis compared to normal cows.

miRNA ID	Fold Regulation	p-value
bta-miR-15b	84.3353	0.001637
bta-miR-17-3p	73.9288	0.000000
bta-miR-16b	55.0650	0.003596
bta-miR-148a	51.7348	0.004212
bta-miR-26b	41.2998	0.000002
bta-miR-101	32.4032	0.000001
bta-miR-29b	16.7149	0.002533
bta-miR-27b	11.2595	0.000150
bta-miR-215	11.0660	0.000049
bta-miR-18b	9.1139	0.000814
bta-miR-22-5p	7.7978	0.015375
bta-miR-218	5.5139	0.037113
bta-miR-145	4.7176	0.000207
bta-miR-106a	2.3103	0.008010
bta-miR-139	2.1039	0.011376
bta-miR-142-5p	2.0821	0.024574
bta-miR-148b	−31.7115	0.006327
bta-miR-199a-3p	−30.4197	0.001416
bta-miR-122	−24.7943	0.000000
bta-miR-200b	−24.2000	0.000762
bta-miR-10a	−18.4677	0.000000
bta-miR-17-5p	−4.7963	0.000768
bta-let-7g	-4.2337	0.001229
bta-miR-214	−3.3448	0.025404
bta-miR-31	−3.1864	0.000027
bta-miR-205	−2.9627	0.000000
bta-let-7d	−2.7263	0.036396
bta-miR-192	−2.4656	0.000352
bta-miR-30b-5p	−2.1539	0.043542
bta-let-7a-5p	−2.0236	0.045190

Of 84 bovine-specific well-characterized miRNAs investigated, 16 were greater (p ≤ 0.05; fold ≥2) and 14 were lower (p ≤ 0.05; fold ≤−2) in serum of cows with metritis.

**Table 3:** Top ranked (based on total target score of miRDB) targeted genes for up-regulated miRNAs.

microRNA ID	Fold regulation	Targeted gene
hsa-miR-15b-5p	84.3353	ZFHX4, SYNJ1, CDCA4, NUP50, PAPPA, LUZP1, SLC13A3, UNC80, MTMR3, PTPN4, PHF19, RECK, N4BP1, PPM1A, ZMAT3, SLC9A6, AKT3, BTRC, IPO7, SCN8A
hsa-miR-17-3p	73.9288	KAT7, TSHZ3, MAML3, HNRNPA3, LMLN, RAP2A, FAM168B, KIAA1804, VEZF1, EPHA6, TGFBR1, TRIM59, KIAA0232, SESTD1, ZFHX4, EBF1, SLC40A1, RAB21, CNOT2, ARID2
hsa-miR-16-5p	55.065	ZFHX4, SYNJ1, SLC9A6, IPO7, CDCA4, NUP50, PAPPA, LUZP1, SLC13A3, UNC80, MTMR3, PTPN4, PHF19, ZBTB44, RECK, N4BP1, VEGFA, PPM1A, ZMAT3, RNF144B
hsa-miR-148a-3p	51.7348	SOS2, LDLR, RPS6KA5, B4GALT6, CDK19, ABCB7, SZRD1, ZFYVE26, MXD1, NPTN, USP33, GPATCH8, SIK1, GLRX5, B4GALT5, TIMM23, ADAM22, DDX6, STARD13, INO80
hsa-miR-26b-5p	41.2998	SLC2A13, SLC7A11, FAM98A, SLC45A4, ZDHHC6, PITPNC1, STRADB, RNF6, ZNF608, USP9X, MAPK6, MARK1, CLASP2, SLC25A16, CILP, NAB1, EPB41L3, ADM, ULK2, CIPC
hsa-miR-101-3p	32.4032	MPPE1, MOB4, CACNB2, TNPO1, STC1, ABHD17C, FLRT3, MYCN, TSHZ3, LCOR, C3orf58, SOCS5, ZFP36L2, FZD6, REV3L, FZD4, RORA, TMEM65, ZNF654, FGA
hsa-miR-29b-3p	16.7149	COL3A1, ERCC6, NFIA, TET3, DGKH, BRWD3, FBN1, TET1, ATAD2B, RNF19A, VEGFA, DNMT3A, FBXW9, ELN, COL5A3, HMCN1, TMEM183A, GRIP1, ROBO1, NAV3
hsa-miR-27b-3p	11.2595	EYA4, ST6GALNAC3, AFF4, GSPT1, GPAM, TNPO1, AKIRIN1, DCUN1D4, GAB1, GXYLT1, MIER3, CCNK, ABHD17C, TMCC1, FOXA3, TRIM23, ZDHHC17, UNKL, SLC7A11, PPARG
hsa-miR-215-5p	11.066	EREG, DYRK3, LPAR4, ZEB2, MSN, ARFGEF1, CCNT2, PDP1, GPR22, DICER1, ANAPC10, LIMS1, WDR44, RPAP2, FRMD4B, ARL2BP, STX7, CNGB3, BHLHE22, FGD5
hsa-miR-18b-5p	9.1139	NEDD9, RORA, BBX, MAP7D1, INADL, PHF19, ZBTB47, CDK19, ERI1, DICER1, HIF1A, CTGF, GIGYF1, PHC3, GLRB, NCOA1, TMEM170B, CREBL2, ZNF367, TRIOBP

**Table 4:** Top ranked (based on total target score of miRDB) targeted genes for down-regulated miRNAs.

microRNA ID	Fold regulation	Target genes
hsa-miR-148b-5p	−31.712	DLST, ZC3H14, ETS1, BACH2, UBE3A, C6orf62, SSBP2, TCEAL1, KCNIP1, ELOVL2, FMO3, SHANK2, UBL3, CDK19, ARHGAP31, RGS2, ASF1A, ZBTB44, PGM3, CDCP2
hsa-miR-199a-3p	−30.42	ETNK1, CELSR2, ADAMTSL3, KLHL3, ACVR2A, LRP2, BCAR3, SERPINE2, NOVA1, MAP3K4, FAM110C, KIAA0319L, RB1, ZHX1, KDM5A, PSD2, LIN28B, LLGL2, ITGA3, CHMP5
hsa-miR-122-5p	−24.794	HNRNPU, CLIC5, CLIC4, PIP4K2A, CPEB1, FKBP5, SCN3B, CD40LG, FOXP2, ANKRD13C, P4HA1, COX10, PAPOLA, RABL6, BAI2, C20orf112, TOR3A, LAMC1, SLC52A2, FUNDC2
hsa-miR-200b-3p	−24.002	TCEB1, TRIM33, LHFP, PTPN21, ARHGAP6, VASH2, HIPK3, NR5A2, ZEB2, WASF3, ZEB1, RECK, SLIT2, AP1S2, ERRFI1, AFF3, CCNJ, MAP4K5, SESN1, ELL2
hsa-miR-10a-5p	−18.468	BDNF, KCNA6, RORA, TFAP2C, CSMD1, ZNF367, SON, PATL1, NR6A1, KLHL29, TRIM2, ARHGEF12, RBMS3, GALNT1, RPRD1A, KLF11, LCA5, SLC24A4, BBX, CLCC1
hsa-miR-17-5p	−4.7963	ZNF800, ARID4B, ADARB1, PTPN4, PKD2, GAB1, SLC40A1, ZNFX1, FBXL5, EPHA4, PDCD1LG2, EPHA5, FGD4, EZH1, STK17B, ARID4A, GPR137C, USP3, SALL1, MASTL
hsa-let-7g-5p	−4.2337	SMARCAD1, LIN28B, FAM178A, GATM, LRIG3, GNPTAB, DNA2, TMEM2, BZW1, ADAMTS8, ADRB2, C8orf58, TTLL4, TMPRSS2, IGDCC3, NME6, HIC2, SCN4B, DMD, ZFYVE26
hsa-miR-214-3p	−3.3448	NAA15, SEC24C, ATP2A3, UHMK1, MED19, RNF169, FBXO32, AKAP13, SARM1, FNDC5, TBL1XR1, PIK3CB, HR, SPIRE1, SNX12, PTER, RCSD1, EZH1, TGOLN2, RALGAPB
hsa-miR-31-5p	−3.1864	RSBN1, IDE, PIK3C2A, SEPHS1, PDZD2, TBXA2R, HIAT1, LBH, RFWD3, PRKCE, SH2D1A, SH2D1A, GXYLT1, GCH1, CCAR2, EGLN3, LATS2, CAMK2D, IGFBP7, MAPKAPK2
hsa-miR-205-5p	−2.9627	CCNJ, CLDN11, CDK19, PTPRM, ZFYVE16, CDK14, C11orf86, FOXF1, PLCB1, SLC19A2, SORBS1, BTBD3, EZR, KANSL3, HS3ST1, CALCRL, MLLT4, TAPT1, GPM6A, EVA1C
